# Glucose biosensors in clinical practice: principles, limits and perspectives of currently used devices

**DOI:** 10.7150/thno.64035

**Published:** 2022-01-01

**Authors:** Salvatore Andrea Pullano, Marta Greco, Maria Giovanna Bianco, Daniela Foti, Antonio Brunetti, Antonino S. Fiorillo

**Affiliations:** 1 Department of Health Sciences, Magna Græcia University of Catanzaro, 88100, Catanzaro, Italy.; 2 Department of Experimental and Clinical Medicine, Magna Græcia University of Catanzaro, 88100, Catanzaro, Italy.

**Keywords:** assessment of glycemic control, glucose sensors, biological fluids, diabetes technology, point-of-care testing.

## Abstract

The demand of glucose monitoring devices and even of updated guidelines for the management of diabetic patients is dramatically increasing due to the progressive rise in the prevalence of diabetes mellitus and the need to prevent its complications. Even though the introduction of the first glucose sensor occurred decades ago, important advances both from the technological and clinical point of view have contributed to a substantial improvement in quality healthcare. This review aims to bring together purely technological and clinical aspects of interest in the field of glucose devices by proposing a roadmap in glucose monitoring and management of patients with diabetes. Also, it prospects other biological fluids to be examined as further options in diabetes care, and suggests, throughout the technology innovation process, future directions to improve the follow-up, treatment, and clinical outcomes of patients.

## Introduction

Glycemic monitoring is currently a routinary and simple operation that is performed intensively worldwide 1,2. Behind that, there is a long history of socio-demographic, technological and clinical practice 3-5. The development of glucose sensors has accompanied the evolution of monitoring and treatment of diseases, in particular of diabetes mellitus, substantially improving glycemic control and preventing the rise and progression of diabetes-related complications 6. Although enzyme-based glucose sensors have dominated the scientific research and the market, these systems inherently suffer from low thermal and chemical stability. Further, one time enzyme-based strips are costly, which discourages and limits frequent testing 7,8. To account for these limitations, non-enzymatic sensors, and methodology for glucose monitoring in alternative biofluids, rather than blood, have been developed 9. In recent years, novel materials have also been introduced, but, even with the advent of nanotechnology, the analytic performance of sensing devices is only possible to a certain extent 10. Referring to glucose monitoring, there is still a demand for continuous and non-invasive detection with more reliable and sensitive devices. A debate on the correlation of glucose levels in alternative biofluids (e.g., tears, saliva, interstitial fluid (ISF), sweat, and urine), with respect to blood is still open and actual 11. The skin has become very popular in recent years, so that new approaches/devices have been developed to minimize the invasiveness (e.g., short-term subcutaneous implantable sensors) 12. Non-invasive systems can meet both the patient's needs and the clinical reliability of glucose detection. Studies on the improvement of new non-invasive monitoring systems are likely to continue to grow 4,10,13,14. Furthermore, to achieve a wide use of these new technologies, the final detection device has to be developed at a very low cost to compete with the currently available blood glucose meters 15. Consequently, efforts and new approaches to establish glucose monitoring based on alternative biofluids, whose reliability is comparable to that reached on blood, represent a promise for glucose monitoring in daily routine and an important goal for diabetic health care in the future 16-18. In parallel, the daily clinical research and practice is an incentive for the development of international organizations that deal with the standardization in the use of these devices, whose accuracy is affected by manifold factors (e.g., pre-analytical sampling, sample characteristics and environmental parameters) 19. The rationale behind this review is not simply to cover technological or clinical advances on glucose monitoring in the last years, but also to provide a global view on the topic using a transversal approach, and a combined vision on the evolution of glycemic control. To this end, we provide an overview of the technologies and methodologies for the evaluation and monitoring of glucose in blood and other less explored biological fluids. Furthermore, we discuss advantages and limits of the glucose biosensors currently used in clinical practice, and future directions that may implement glycemic control and clinical outcomes in the light of currently adopted treatments in diabetes care.

### Assessment of glycemic control in diabetes mellitus

Diabetes mellitus (DM) is a widespread, clinically heterogeneous disease characterized by a chronic increase in blood glucose levels due to impaired insulin secretion and/or peripheral insulin resistance 20-23. Insulin-dependent type 1 diabetes accounts for about 5-10 % of all cases of diabetes, and is characterized by a failure in insulin production for an autoimmune attack on pancreatic beta cells 24,25. Type 2 diabetes, also known as “non-insulin-dependent” diabetes, is the most prevalent form of diabetes and is caused by a combination of genetic and environmental factors. In this latter form, the relatively low insulin production, due to a progressive beta cell dysfunction, frequently combines with a background of insulin resistance at the level of skeletal muscle, liver, and adipose tissue 26,27. The most recent classification from the American Diabetes Association (ADA) includes other less common forms of diabetes, in addition to gestational diabetes mellitus, which can develop during pregnancy and usually resolves after delivery 28. Regardless of the type of diabetes mellitus, many affected people develop a number of acute (diabetic ketoacidosis, hyperosmolar hyperglycemic nonketotic coma, severe hypoglycemia) and chronic microvascular (diabetic retinopathy, nephropathy and neuropathy), and macrovascular (coronary heart disease, cerebrovascular disease, and peripheral vascular disease) complications that are the leading cause of morbidity and mortality among these patients 29-31. Hypertension, dyslipidemia and heart failure, which are primary predictors of cardiovascular mortality, are also significantly more common in diabetic than in non-diabetic individuals 31,32.

Glycemic control is crucial to prevent the rise and progression of diabetic complications 31,33, so that glycemic targets have been proposed in various specific settings 34. Currently, the assessment of glycemic status can be carried out by the measurement of glycated hemoglobin (HbA1c), the assessment of self-monitoring blood glucose (SMBG), and the use of continuous glucose monitoring (CGM). HbA1c estimates the average blood glucose levels over approximately 3 months and is recommended 2-4 times a year, depending on patient's treatment goal. SMBG is indicated for self-management and pharmacological adjustments, especially in patients under insulin treatment, while CGM plays an important role in both prevention of hypoglycemia and therapy's effectiveness and safety in patients with type 1 diabetes or in selected cases of insulin-treated type 2 diabetes 35.

Even though a variety of predictive, diagnostic, and prognostic biomarkers (and related technologies) are continuously proposed, blood glucose concentration and HbA1c are still the major biomarkers for both diagnosis and patient monitoring 36,37. The direct costs related to the management (diagnosis, monitoring, treatment, etc.) of diabetes accounts for billion dollars annually, besides the indirect costs from missed work or decreased productivity 38. Since the invention of the first enzymatic electrode in 1962 39, many efforts have been made to improve the performance of glucose sensors technology. Today, the development of devices for glucose monitoring with high reliability need new technologies and strategies to make those sensors more affordable, non-invasive, and suitable for continuous monitoring of glycemic status 40-42.

### Evolution of glycemic monitoring

**Figure 1:**
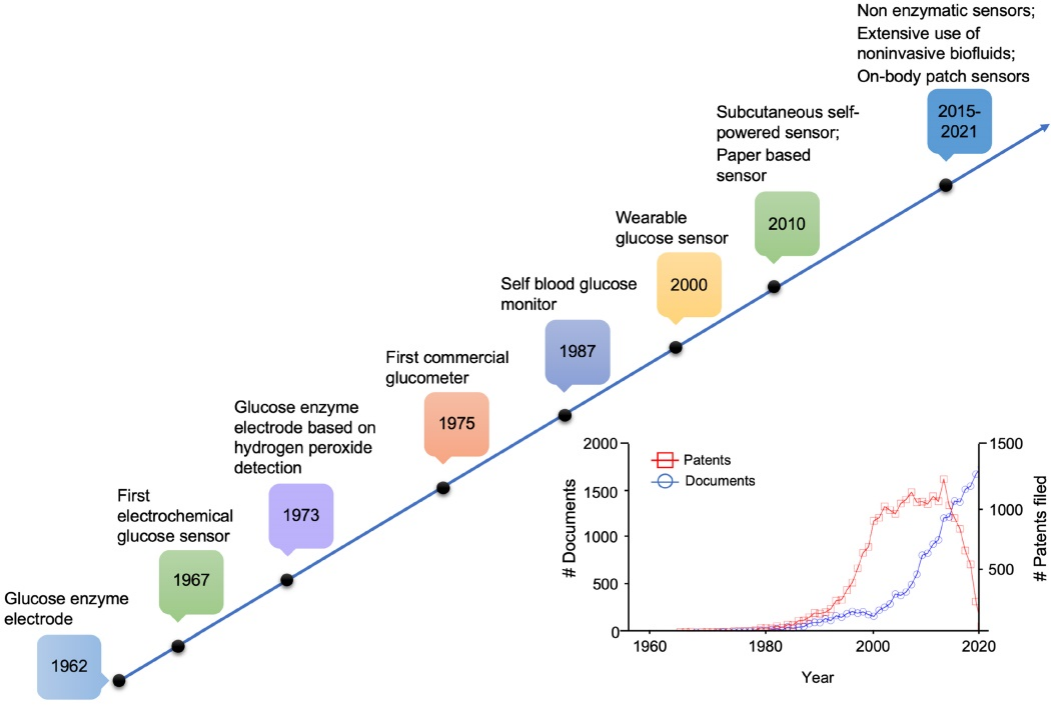
Milestones in the development of actual glucose sensor systems technology. In the inset, available literature and estimated patents filed involving glucose sensors (1955-2020). Sourced from Scopus (blue) and Google (red).

The concept of a glucose sensor was firstly proposed in 1962 by Clark and Lyons, who described an amperometric electrode for the determination of blood glucose through an enzymatic method, using glucose oxidase (GOx or GOD) 39. This system represented an evolution of a previous electrode proposed by Clark for the determination of oxygen, intended to be mounted on an intravascular catheter 43. The GOx enzyme catalyzes the oxidation of glucose, leading to the formation of hydrogen peroxide, so that a decrease in the oxygen concentration is proportional to glucose concentration 44. However, the overall mechanism is strongly influenced by the background oxygen level, that adversely affects sensor's accuracy. In 1967, an electrochemical biosensor was proposed by Updike and Hicks using enzymes immobilized in a polyacrylamide gel on the surface of an oxygen electrode working in single or dual mode (single or dual cathode) 45. In a dual cathode configuration, one electrode is coated with the active enzyme and the other with the same enzyme, but inactive (i.e., unresponsive electrode), to reduce the above-mentioned limitation of oxygen background concentration 45. In 1973, Guilbault et al. described a glucose biosensor based on the amperometric monitoring of hydrogen peroxide originated from enzymatic catalysis 46. Only in 1975, a first sensor for the direct glucose measurement was proposed. In fact, the technology developed by Clark and Lyons was transferred to Yellow Spring Instrument Company (YSI), which presented a whole blood analyzer, Model 23 (a platinum electrode mainly for clinical use due to its cost). As shown in the timeline in Figure 1, starting from the 1960s onwards, the development and application of glucose sensor in the medical sector has aroused considerable interest in both the academic and industrial fields.

The above-mentioned sensors belong to the “first generation” of glucose sensors, which exploit an oxygen electrode, acting as a substrate, and the production of hydrogen peroxide. Intense efforts during the last decades have led to the development of the so-called mediator-based “second generation” of glucose sensors 47,48, the introduction of commercial strips for SMBG 49,50, and the use of further modified electrodes for enhancing the performance 51. Advancements were obtained by replacing oxygen with a non-physiological (synthetic) electron acceptor (mediator) capable of transporting electrons from the center of the enzyme to the electrode surface. The transfer of electrons between the enzyme active site and the electrode surface is the limiting factor in the functioning of the amperometric glucose sensors 47. These mediator-based sensors increase the rate of electron transfer between the electrode and the enzyme. Further studies were focused on the development of a technology that did not need any mediator to obtain a reagent-free glucose sensor. The “third generation” of such sensors is based on reagentless devices, and therefore on the direct electron transfer between the enzyme and the electrode without the use of mediators, which are usually toxic. The main advantage is the high selectivity since the working potential is identical to that of the enzyme and, therefore, less prone to any interference 47,48. More recently, devices based on the direct electro-oxidation of glucose are being proposed as a possible “fourth generation” of sensors, which mainly use noble metals as catalyst to overcome the limits of enzymatic sensors 49. Despite the target is the glucose detection in complex sample matrix (tears, saliva, ISF, sweat and urine), in most cases, equivalent samples were investigated for glucose performances. Hereafter, literature review is mainly limited to biological samples, which can provide reliable clinical outcomes.

### Sensor reliability

Whether they are in development or already in clinical practice/home environment, concerns about the reliability of sensors are still cause of discussion. The gradual evolution of the glycemic monitoring provides results in a few seconds from only 0.3-1 μL of blood (e.g., SMBG) 50-52. Therefore, the accuracy of glucose sensor (often referred to glucometer) represents still today a debated issue. In fact, glucose levels in the same sample should ideally be compared to a reference or comparative method. Unfortunately, this is technically difficult due to the small volume of capillary blood that can be obtained. This process is also cumbersome because the glucometer measures a whole blood sample, in which glucose levels are unstable due to glycolysis. In fact, blood cells, and in particular erythrocytes, metabolize glucose and may progressively reduce its concentration at a rate of 5-7 %/h as long as plasma remains in contact with the erythrocytes 53. Therefore, the use of whole blood samples for accuracy comparisons requires consideration of the effects of glycolysis and separation of plasma from the cells for laboratory analysis within a reasonable period (generally within 30 min). However, accuracy comparisons are often conducted on a capillary sample analyzed by a glucose meter versus a venous plasma sample collected at the same time and analyzed with a laboratory method (e.g., venous samples collected in lithium heparin containing tubes) 54,55.

**Figure 2:**
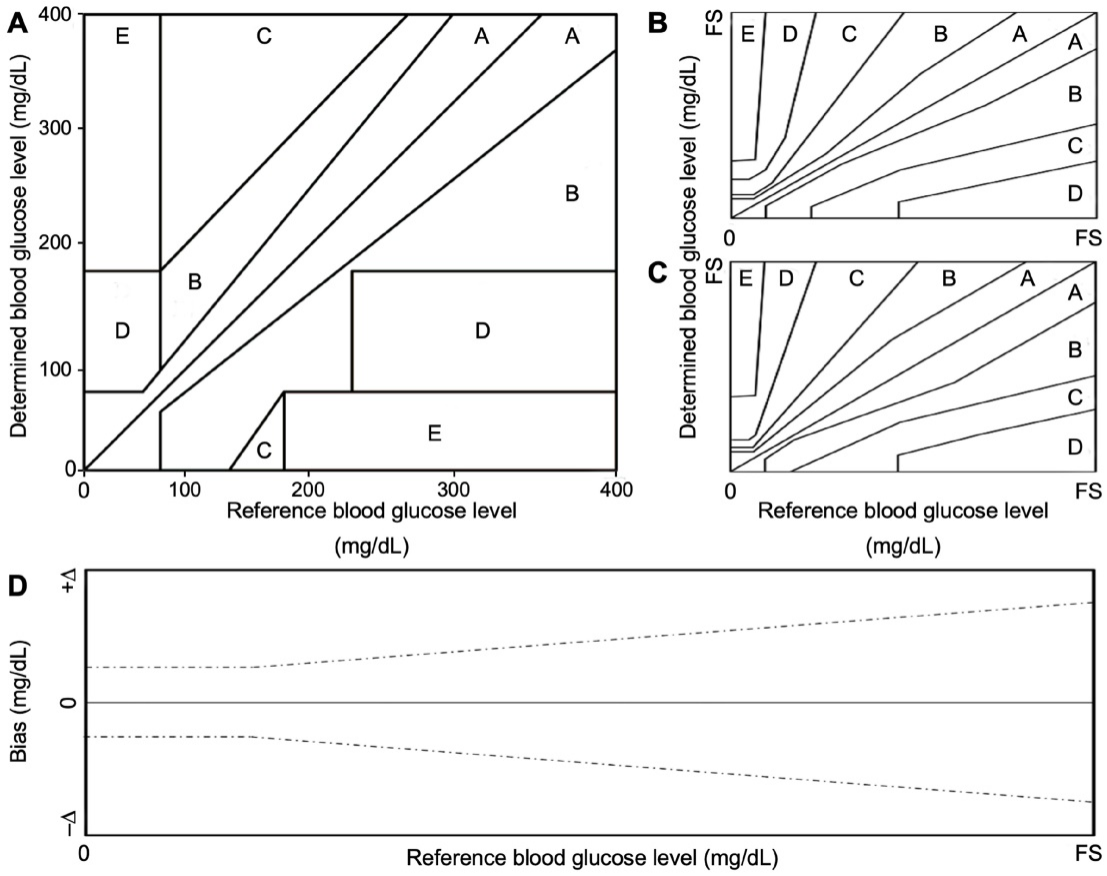
Error grid analysis proposed by Clarke et al., for clinical accuracy (A), and further modified by Parkes et al., for type 1 (B) and type 2 (C) diabetes. System bias plot (D), dashed black lines indicate the predetermined accuracy limits. FS represents the full-scale level for glucose concentration of the tested and reference sensor. Classically, it is set at 400 mg/dL (blood-based sensors).

In 1987 Clarke et al. attempted to address clinical agreement by developing an error grid that evaluated the clinical significance of the determined glucose level of a device under test, versus a gold-standard method (Figure 2A) 56. Clarke error grid is divided into 5 precision zones. A slight deviation (< 20%), or results in the hypoglycemic range (< 70 mg/dL) between the two methods fall within zone A. B zone is characterized by a higher deviation (> 20%) and would not lead to changes in the clinical decision. Conversely, significant differences from the gold-standard method fall in zones C, D or E. The first is characterized by overcorrected acceptable results, while zone D and E represent “potentially dangerous errors to detect and treat” and “erroneous treatment”, respectively 56. In 2000, this grid was further modified by Parkes et al., for type 1 and type 2 diabetes (Figure 2 B, C, respectively), to avoid discontinuity between risk areas, in which small changes in glucose levels could lead to significant changes of clinical impact (Parkes error grid or consensus error grid) 57. The above-mentioned analysis was proposed for “clinical accuracy” purpose, to quantify the probability of making a correct therapeutic decision based on the obtained result with a glucometer. Analytical accuracy, instead, is evaluated by comparing the bias between the result provided by the device under test and a gold standard method (specified by the manufacturer) (Figure 2D) 57,58.

Standard organizations and scientific societies differ on the acceptability criteria for accuracy, so there is no single standard for evaluating the accuracy of a blood glucometer. Regulatory organisms worldwide provide and continuously update standards describing the requirements for blood glucose monitoring systems for health care professionals and lay users 59-65. Table 1 refers to the main standards developed by international organisms for standardization 60-64.

The analytical performance of the instrument also depends on what purpose the information obtained is being used for: screening, diagnosis, or management. Upon proper comparison, it may be discovered that glucometers should not be used to diagnose diabetes but may instead be suitable for patient monitoring and insulin management 66. There is open debate on how international standards should be applied and improved when comparing glucometers for automonitoring and laboratory devices 67-73. Sensors must have certain characteristics to be efficient and of quality. In 1994, the ADA made the first recommendations for the analytical performance of glucose biosensors available, suggesting a threshold <10 % of maximum permissible bias from reference methods for glucose concentrations between 30 and 396 mg/dL. This analytical target was further reduced to <5% in 1996 52. According to the Food and Drug Administration (FDA) recommendations, glucose sensors must have an error <20% for glucose concentrations between 30 and 396 mg/dL compared to reference laboratory measurements. The intermediate accuracy of a sensor is defined as accuracy under conditions where test results are obtained by the same method on the same type of sample, in the same position, but where other variables such as operators, equipment, calibration, environmental conditions and/or time intervals differ. Repeatability is evaluated at five diffuse glucose concentrations in the measurement range (30-50 mg/dL, 51-110 mg/dL, 111-150 mg/dL, 151-250 mg/dL, and 251-400 mg/dL) and should be measured over a short period of time, with the same group of users, meters, and reagents. The preferred sample is venous blood. The evaluation of all these, and other parameters allow the evaluation of the technical precision of the device under examination; in the case of biosensors, it is then necessary to complete the study of the functionality of the instrument alongside the evaluation of the clinical precision of the sensor, which consists of comparing the results obtained on a sample by using the sensor in question with a standard laboratory method. The data can be entered into the Clarke or Parkes error grid to allow clinicians to make the right decision without compromising patient's health.

### Classification of glucose sensors

Electrochemical glucose sensors can be divided into potentiometric (employed to detect variations of surface charge onto a counter electrode), amperometric (charge flow between the counter electrode and the bio-system), or conductometric (variations in ionic conductance between electrodes) 74,75. As stated above, over the years, enzymatic amperometric glucose sensors were the first and widespread glucose sensors available 76. They are generally fabricated by using two families of enzymes, the glucose oxidase and the glucose dehydrogenase (GDH). The reaction, catalyzed by the GOx, is as follows:



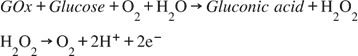



**Figure 3:**
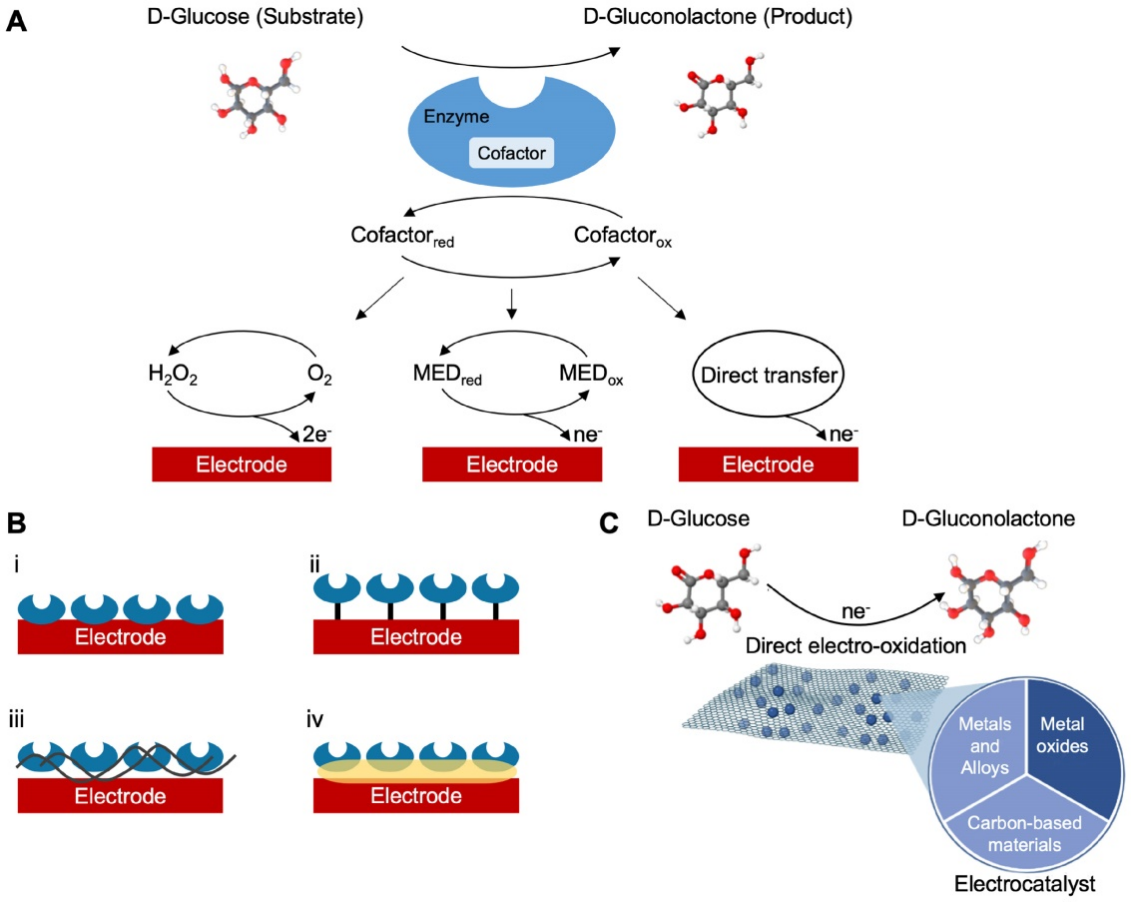
(A) Schematic classification of the glucose biosensors evolution distinguished into generations according to the sensing mechanism. (B) Representative enzyme immobilization techniques: i, adsorption; ii, covalent bonding; iii, cross-linking; iv, entrapment. (C) Schematic representation of a characteristic direct electro-oxidation of glucose in non-enzymatic glucose sensors and the most investigated materials used as catalyst.

In many complex sample matrices such as blood, the dextrorotatory enantiomer of glucose (D-glucose) equilibrates among α-D-glucose, β-D-glucose structures (> 99.9%), and aldehyde form. Since GOx is inherently highly selective for β-D-glucose, preparations require the interconversion among these forms (enzyme mutarotase, phosphate ions etc.). Since GDH is also selective for β-D-glucose, the same reaction can be catalyzed by replacing GOx. The concept behind a glucose biosensor is based on the fact that the immobilized GOx catalyzes the oxidation of D-glucose by molecular oxygen producing gluconic acid and hydrogen peroxide (Figure 3A). To function as a catalyst, GOx requires a flavin adenine dinucleotide (FAD) redox cofactor. FAD functions as an initial electron acceptor and is reduced to FADH_2_ 76. This enzymatic electrode evaluates the glucose level by the amperometric tracking of the released hydrogen peroxide 76. Glucose dehydrogenases are instead defined as oxidoreductases which are unable to use oxygen as an electron acceptor and therefore transfer electrons to other natural and artificial acceptors. GDHs also need a cofactor. These are mainly nicotine adenine dinucleotide (NAD^+^ or NADH depending on the oxidation state) or pyrroloquinoline quinone (PQQ) 45. FAD, NAD^+^ and PQQ remove hydrogen, H^+^ and e^-^, from glucose according to the following:



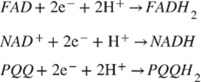



Natural acceptors can be replaced by artificial electron acceptors such as ferrocene and its derivatives, phenazine methosulfate (PMS), or phenazine ethosulfate. The pure carbon surface is also an improper material used as an electron acceptor from the active center of the enzyme 77. GDH-PQQ is a particularly efficient enzyme system, with a fast electron transfer rate, but it is relatively expensive. GDH with NAD^+^ as a cofactor produces NADH rather than H_2_O_2_. Nicotine adenine dinucleotide is an important electron acceptor in glucose oxidation, during which NAD's nicotinamide ring accepts one hydrogen ion and two electrons, equivalent to one hydride ion. In this reaction, the generated reduced form of this cofactor is NADH, which can be electrochemically oxidized. As previously introduced, based on the use of specific enzymes and co-factors, glucose sensors can be classified as reported in Table 2.

An electrochemical biosensor is composed by working electrodes (on which the reaction of interest, responsible for the measurement, takes place), reference electrodes and auxiliary electrodes (to ensure that the current does not circulate through the electrode). Glucose concentration is mostly evaluated using the amperometric method, that monitors the current flowing between the working electrode and the reference electrode. The latter involves the application of a potential, that in turn results in the contribution of other electroactive species (e.g., ascorbic acid, uric acid) reducing the selectivity of the electrode. Another limiting aspect of the enzymatic glucose sensor (especially those of the first generation), is due to the oxygen deficit inside the sample. To this end, the use of additional membranes, mediators, and electrocatalysts were investigated 78. The role of mediators is crucial in glucose electro-oxidation. For example, FAD-GOx provides a low-rate of oxidation and thus the use of mediators allows a rapid glucose oxidation, giving reliable results. Different families of materials were used as redox mediators for FAD-GOx, PQQ-GDH, and NAD-GDH electrodes, such as those of ferrocene derivatives (e.g., ferrocenecarboxylic acid, ferrocenemethanol), osmium complexes (bis-(2,2'-bipyridine) osmium(II), bis-(4,4'-dimethyl-2,2'-bi-pyridine) osmium(II)), ruthenium (ruthenium hexamine), and organic mediators (quinone derivatives) 79,80.

The optimal performance of the electrodes within a sensor requires the choice of suitable materials in relation to the kind of enzymatic reaction taking place. In addition, the selectivity and sensitivity of the reaction strongly depend on the characteristics of the working electrode. In general, sensitivity can be modulated by adjusting the surface area of the working electrode 81. Porous electrodes, for example, lead to higher sensitivity than planar electrodes because porous electrodes have a larger surface area to accommodate the chemical reaction. An inherent sensitivity to environmental conditions (pH, temperature, humidity and chemical condition of the sample) is one of the bottlenecks of the enzyme-based technology, which reduce the stability of the sensors, especially in those applications in which environmental conditions are not controllable (continuous glucose monitoring, wearable devices). Another factor that determines the quality of the electrode in glucose measurement is the immobilization of the enzyme on the electrode: the permanent immobilization leads to reliable and long-term performance. For this to happen, the enzyme is cross-linked with hydrogel (e.g., chitosan and gelatin), nanomaterials (e.g., carbon nanotubes and graphene) and other stabilizers (e.g., bovine serum albumin) by chemical and physical bonds. Table 3 shows a non-exhaustive list of materials used for the fabrication of working electrodes, the reference enzyme (GOx) to catalyze the reaction and the immobilization technique (Figure 3B) 7,76,77.

Apart from the above reported amperometric biosensors, reference method for the clinical determination of glucose using spectrophotometry in many laboratories is based on hexokinase (HK) assay. In this case HK catalyses the phosphorylation of glucose (glucose-6-phosphate) using adenosine triphosphate (ATP), becoming adenosine diphosphate (ADP). Bacterial glucose-6-phosphate dehydrogenase (G-6-PDH), in the presence of Nicotinamide adenine dinucleotide phosphate (NADP), causes the oxidation of glucose-6-phosphate to gluconate-6-phosphate. The production rate of NADPH is directly proportional to glucose concentration (and is measured photometrically at 340 nm), according to the following reaction:



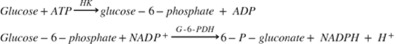



This test is a reference method for the quantitative determination of glucose in serum, in human plasma, urine and cerebrospinal fluid 52.

The novel concept of nonenzymatic sensors is being proposed for their foreseen performances in terms of stability (some evidence report sufficient stability up to 30 days in complex samples), easier fabrication process (Figure 3C), besides the very high sensitivity and the variety of sensor materials that are currently investigated 49. Obviously, the design of glucose sensors should consider parameters such as sensitivity and limit of detection (LOD), especially in relation to the biofluid used to identify the best suited technology. Non enzymatic sensors based on metal, metal-oxide, or based on carbon (carbon nanotubes, graphene) are characterized by a higher sensitivity with respect to enzymatic counterparts 82-86. For example, non-enzymatic sensors based on carbon nanotubes decorated with Nickel evidenced a sensitivity of 70 mA mM^-1^cm^-2^, which is outstanding if compared with GOx based sensors with a sensitivity that, in most cases, is different order of magnitude lower 87. A similar conclusion can be observed considering LOD, even though, it evidenced a higher degree of variability, non-enzymatic sensor reaches more often LOD on the nM range. Despite a vast scientific literature on non-enzymatic glucose sensors has been produced in the last years, the technology has not reached the commercial phase and thus clinical experience is still limited. Sensor stability represents a major concern in CGM devices, which are expected to provide reliable data for a sufficient timeframe (the devices currently on the market are indicated for 7 days of use requiring multiple calibrations per day). However, even though invasive, extended time CGM based on a subcutaneous approach is able to provide accurate measurement for up to 1 year. The latter, generally requires 2-3 weeks stabilization period after surgery and are larger than the subcutaneous counterparts 88. Despite recent data from nonenzymatic sensors evidenced a higher sensitivity with respect to enzymatic counterparts, they still suffer of a poor stability (i.e., surface fouling of the electrode) and lacks glucose selectivity 49. It is widely known that enzymatic sensors such as those based on glucose oxidase are more stable than other enzymes, easy to obtain, and cheap; on the other hand, it quickly loses its activity at pH<2 or pH>8 and can be irrevocably damaged at temperatures above 40 °C 76. Also, it is affected by environmental unstable humidity before its use 77. Despite the chief motive of this review is to provide a multidisciplinary approach for a wide audience in the technical/clinical field, being a highly attractive area, a vast literature is witnessed by systematic readings that can be reached to deepen technological advancements on enzymatic, nonenzymatic, and optical based glucose sensors 4,9,49,50,51,75,76,78,79,88.

**Figure 4:**
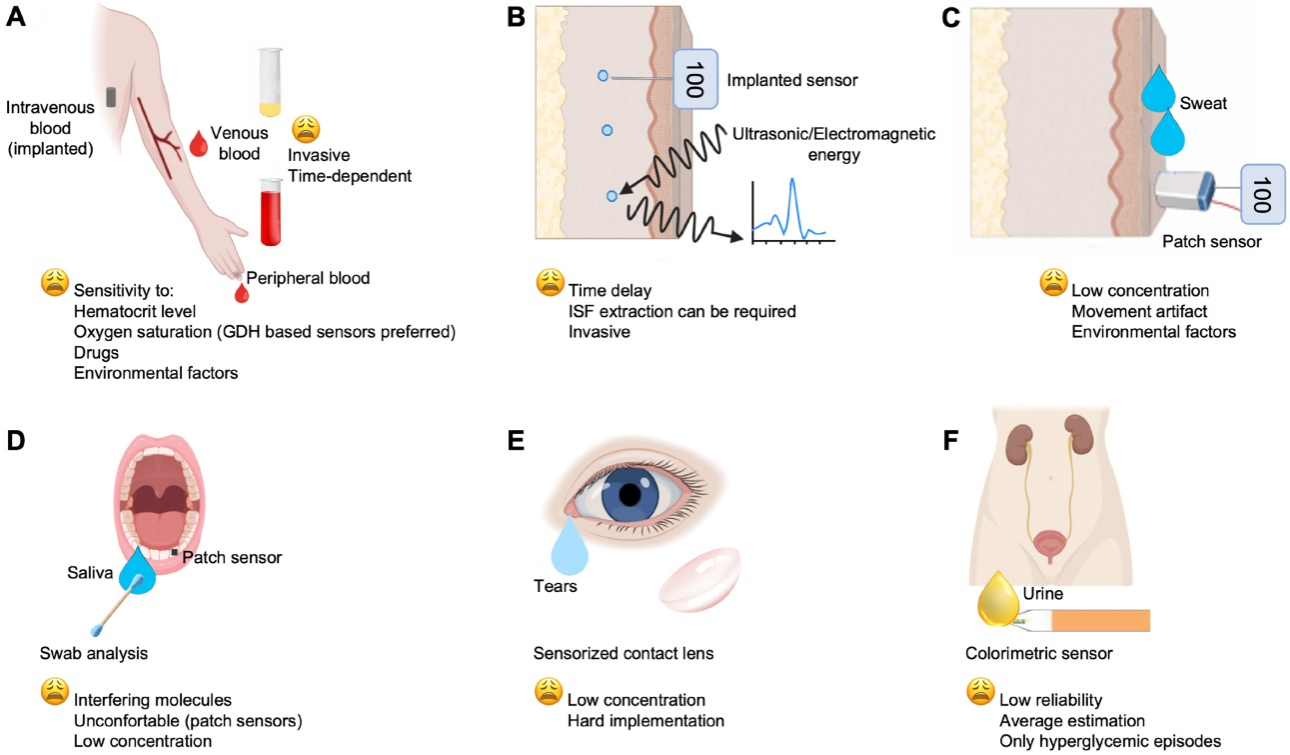
Main biofluids and technologies investigated for glucose monitoring, which include: (A) the gold standard venous blood and the widespread used peripheral blood for auto-monitoring; (B) ISF; (C) sweat; (D), saliva; (E) tears; (F) urine.

Another way to disentangle the variety of devices developed/commercialized can be changing the sample type used. Venous and peripheral blood glucose concentration (Figure 4A) is the main basis for the diagnosis, monitoring and treatment of diabetes, but for auto-monitoring, other biological fluids can be also used for glucose determination; for example, ISF, sweat, tears, saliva and urine. Currently, the World Health Organization (WHO), has established the expected values for fasting blood glucose (FBG) of normal, euglycemic people, at 70-100 mg/dL, and the blood glucose after 2 h from oral glucose tolerance test (OGTT) at < 140 mg/dL (https://www.who.int). Indeed, it is remarkable that glucose levels in biofluids, such as ISF (Figure 4B), sweat (Figure 4C), saliva, tears, and urine (Figure 4 D, E, F, respectively) are correlated with those in the blood 76. Therefore, many studies have focused on these biofluids to develop noninvasive sensors and methods for glucose monitoring. Most importantly, glucose level in these fluids is lower than that in the bloodstream. Its concentration range varies between 36-720 mg/dL in blood, 36-400 mg/dL in ISF, 0.0001-32 mg/dL in saliva, 0.00018-20 mg/dL in sweat and 0.0009-90 mg/dL in tears 11. Hereafter, glucose monitoring is described according to the site of measurement and the applied technologies.

### Blood glucose monitoring

Most of the gold standard tests for clinical diagnostics exploits the use of blood, and glucose is no exception 89. However, blood sampling is invasive and expensive for a large number of tests per day. Alternatively, implantable sensors can be used for continuous glucose monitoring. SMBG mainly refers to the monitoring of peripheral blood glucose concentration in a specific time of sampling 90. Blood sampling at capillary level results in higher glucose concentration than in venous blood and is affected by the metabolic state 91. Currently, there are different commercial SMBG (e.g., Roche, Sano, Omron, Johnson and Johnson, Bayer, Abbott, Echeng, Ecco, etc.) (Table 4) 92-97 Apart from the invasive blood sampling, the portability, and the relative simplicity of operation, together with their relative high accuracy, have allowed a wide diffusion of these devices. However, being used multiple times a day, their relative cheapness is apparent only.

### Role of the pre-analytical phase

Factors affecting accuracy of results may occur in any step of the diagnostic process. In this context, pre-analytical aspects are of crucial importance 98. Operators should standardize the use of SMGB by reactivating circulation in the finger chosen for the puncture by massaging the hand from the palm to the fingertips; hands need to be preferentially washed with water and soap and dried; an appropriate lancing device and puncture depth should be set. The first blood drop is generally considered the best biological matrix to be used 99. Milking the finger, instead of massaging, should be avoided. Opened or expired test strips can also represent a source of SMBG inaccuracy and should not be used.

### Glucose sensor inaccuracy in specific clinical settings

Many variables, including hematocrit, hypoxemia, hypotension, temperature, altitude, and humidity may influence the reliability of glucose measurements 52,100. In strip-based glucose assays, high hematocrit values, due to increased blood viscosity, can reduce the diffusion of plasma and determine underestimated results, whereas low hematocrit levels, like in anemia, are associated with overestimated readings 101,102. Most glucose biosensors are, therefore, reliable only if hematocrit value is not far from the normal range.

Glucose oxidase-based devices are sensitive to oxygen and should be only used with capillary blood from patients with normal oxygen saturation. In case of higher oxygen tension (oxygen therapy or arterial blood), glucose readings are falsely low, while in case of lower oxygen tension (hypoxia, high altitude, venous blood), test results are falsely high 100,103. Therefore, for patients in critical care, Point-of-Care Test (POCT) should prefer the use of glucose dehydrogenase-based monitors since they are not sensitive to oxygen. These latter devices are not recommended in patients on peritoneal dialysis, in which the osmotic agent icodextrin, a widely used glucose polymer, may cause falsely elevated glucose readings and improper insulin administration in patients with diabetes 104,105. In the case of glucose oxidase-based monitors, physiological and pharmacological interfering substances for glucose readings include uric acid, galactose, xylose, L-DOPA, acetaminophen, and ascorbic acid 100,106,107.

### Glucose monitoring in interstitial fluid

Glucose levels can be monitored in the extracellular fluid that surrounds tissue cells, i.e., ISF (Figure 4B). In tissue, the cells are not directly in contact with the capillaries, while exchanges between blood and cells are mediated by ISF, thus allowing the passage of electrolytes, nutrients, and waste, as well as hormones 98. Glucose measured in the ISF represents a good indicator of blood glucose level due to the continuous supply of this nutrient from vessels to the interstitial area. The diffusion of glucose from the capillary to the ISF occurs, however, with a short delay of 5-10 min, thus limiting the reliability of results in hypoglycemic emergencies 99.

Iontophoresis or reverse iontophoresis is based on the injection of current through the measurement site, which causes migration of ions and glucose from the ISF to the surface and electrodes 100. Ion migration generates the charge flow, while the glucose (uncharged) is transported exploiting the current, by convective flow 101. Thus, it is collected in the passage through the skin, at the cathode where a glucose biosensor is placed. Glucose extraction is approximately in linear relation with the density and duration of iontophoretic current (<0.5 mA/cm^2^) 102. The main drawback of this technique is that it may cause skin irritation, mainly in case of longer exposures to iontophoretic treatment. On the other hand, a minimum duration is required to get enough glucose for measurement 103,104. Sweat represents an interference factor. Also, concerns are raised to whether this technology can reflect rapid changes in blood glucose (common to all the non-blood glucose sensors). Sonophoresis involves low frequency ultrasonic wave in order to increase skin permeability and have access to ISF 105. Extracted ISF can be easily analyzed externally by optical/electrochemical glucose biosensor 106. In-vivo microdialysis is an important technique in CGM devices; glucose is sampled from the body by a subcutaneous probe consisting of a semipermeable hollow fiber. The membrane surface of the probe is biocompatible and safe for the patient. The sensing electrodes, located in an external unit coupled to the probe, are exposed to a relatively clean sample solution, which makes them less prone to biofouling and more accurate than implantable needle biosensors 107. Although results provided by microdialysis are sufficiently precise, this technique is not yet commonly used due to the high costs of the instrumentation. Furthermore, the instruments are bulky and cannot be worn during daily activities 108. Dexcom G6 (Dexcom), Guardian REAL-time (Medtronic) and FreeStyle Navigator (Abbott) are examples of few minimally invasive devices, which are currently available in the market. These devices use subcutaneous sensors to determine glucose concentration in ISF. In optical devices, glucose concentration is evaluated through the interaction with light (reflection, absorption, scattering) and represents a viable and investigated approach. Near infrared (NIR) spectroscopy is a method that uses a light beam with a wavelength in the near-infrared range of the electromagnetic spectrum (750-2500 nm). When infrared radiation hits a molecule in patient's skin, it releases energy and the molecule vibrates (stretching or bending vibration), producing an oscillating magnetic field due to changes in its dipole moment. NIR spectroscopy allows the measurement of glucose in tissues with a depth of 1-100 mm. It uses three basic measurement modes: transmittance, reflectance (including diffuse reflectance), and interactance. The light beam is partially absorbed and then diffused due to the interaction between light and chemical components present in the tissues. Glucose concentration is estimated as variations in the intensity of the light transmitted and reflected in a tissue 103. The major problem with non‐invasive optical sensors is the specific measurement of glucose with sufficient analytical quality 109.

A complimentary alternative to NIR is represented by Raman spectroscopy, which was recently proposed as a non-invasive glucose sensing technique, even though the commercial application is still to be demonstrated 110. The LOD in this case is in the mM range, which together with the low sensitivity, has required the need for a robust chemometric analysis 110,111. It was investigated as non-invasive tool in ISF and in different other tissues and, in particular cases, Raman spectroscopy was applied to anterior aqueous humor since it is correlated with blood glucose, providing at the same time few optically active (interfering) molecules 110-114

Optical coherence tomography (OCT) scans the biotissues with low coherent light source. The refractive index mismatch (Δn) between cells and ISF and the scattering properties in the dermis, gives information about glucose concentrations according to the principles of low coherency interferometry. OCT can typically produce an image at a tissue depth of up to several mm with a very high resolution (<10-15 μm). The dispersion properties of the tissue are highly dependent on the ratio between the refractive index of the diffusion centers (cellular components, proteins, etc.) and that of the ISF. An increase in the concentration of glucose in the ISF causes an increase in its refractive index, thus determining a decrease in the dispersion coefficient. Therefore, starting from the OCT data, generated by the backscattered light, it is possible to obtain an estimate of glucose concentration in the ISF. However, OCT is sensitive to motion artifact due to the inhomogeneity of tissue 115,116.

Photo-acoustic spectroscopy combines a light emitted from a laser with the acoustic response produced. The ultrasonic transmitter measures the peak-to peak variation of pressure waves due to absorption. First in-vivo scientific evidence highlighted greater sensitivity in the determination of glucose than other more traditional spectroscopic techniques and this is due to the relatively poor photoacoustic response of water, which facilitates the determination of some compounds, such as glucose and hydrocarbons 117,118.

Fluorescence is based on the generation of fluorescence by glucose molecules in blood when excited by lights at specific frequencies (308 nm). Fluorescence intensity is dependent upon glucose concentration, and glucose levels in tears reflect concentrations similar to those in blood 119. There are few fluorescence-based glucose detection methods that have reached the stage of testing *in vivo*, and none has been approved for clinical practice in diabetes management. Spectroscopic techniques are inherently characterized by a higher stability (>30 days), paid by a lower sensitivity and LOD. However, when the analysis is mediated by the presence of an external substrate (e.g., Surface enhanced Raman spectroscopy, fluorescent nanomaterials etc.), the stability can be negatively influenced 120-122. Although a large literature is present on non-invasive glucose monitoring, the research of novel non-invasive techniques is still in progress. Table 5 provides a summarization about the reported technologies 103,104,110-114,117-119,123-145.

### Glucose monitoring in sweat

Sweat-based glucose sensors offer a further theoretical alternative to non-invasive methods for glucose measurement (Figure 4C). However, attention must be paid on the complex, and variable chemical composition of sweat. For example, sweat collection and detection vary, depending on the environmental conditions. In addition to the difficulty of measuring glucose levels in sweat due to its much lower concentration than in blood, lactic acid content in sweat, changes in environmental temperature and various medications can induce errors in enzyme-based glucose detection. Mechanical friction and deformation of the devices on soft human skin might delaminate the enzymes from the sensor, causing mechanical fractures that could affect the glucose estimation 146. An innovative approach to sweat-based glucose measurement was investigated by Saraoglu et al., based on the combined use of humidity sensor and artificial neural networks (ANN). A comparison of glucose measurements obtained in sweat and in blood showed a relative error ranging between 2.90%-15.81% and 5.13%-16.25%. These relative errors were established for blood glucose measurements from human palm perspiration 147. Gao et al. pioneered another example of a fully integrated flexible sensor array platform (FISA) for *in situ* sweat analysis, able to measure multiple sweat metabolites (glucose and lactate) and electrolytes (sodium and potassium), as well as skin temperature in a wearable patch-type platform (patch) 148. It was proposed that the increase in sweat rate and duration led to the dilution of sweat glucose over time, which corresponded to an observed increase in skin temperature. However, different parts of the body showed different rates of sweat, which led to varying concentrations of analytes at a given time due to the dilution effect. Therefore, the study indicated that careful assessment of sweat composition and environmental parameters is required for accurate blood glucose monitoring 149. Wang et al. developed a non-invasive, wearable glucose monitoring platform in the form of an amperometric glucose sensing tattoo on a flexible substrate comprising iontophoretic and glucose sensing electrodes 149. Although the tattoo-based device was designed for single-use, such a sensor offers considerable promise for continuous blood glucose monitoring in the non-invasive ISF by offering a body-compatible, flexible, and cost-effective platform 150. Similarly, there are also a series of patch sensors that are used to assess blood glucose via sweat (or via ISF, but with a minimal invasiveness caused by microneedles) for a day. However, the requirement to sweat whenever a measurement is to be made may be inconvenient or impractical for many potential users 151. Table 6 provides a non-exhaustive summarization above the reported technologies 18,147,148,152.

### Glucose monitoring in saliva

Saliva can be collected in a non-invasive way, without the need for specific equipment or trained personnel. Since saliva collection requires fewer skills than blood collection, its analysis is more valuable for children and the elderly. Saliva analysis can also provide a cost-effective approach for screening large populations 153. However, various impurities in saliva from ingested food and digested metabolites can hinder accurate measurement of glucose concentration (Figure 4D). In general, glucose in saliva can be measured after filtering large biomolecules mixed in saliva 154. Glucose moves easily from plasma through the membranes of the blood vessels to the gum fluid, through the gingival sulcus, reaching saliva. Therefore, hyperglycemia in diabetic patients could lead to higher salivary glucose, whose concentration at this level is typically in the range of 0.5-1 mg/dL, thus much lower than glucose in the blood 155-158. Recent developments in highly sensitive materials are paving the way for generating easy and low-cost methods for acquiring salivary glucose (see Table 7) 155-158. For example, Macaya et al. developed electrochemical sensors using a transistor with a channel consisting of poly (3,4-ethylenedioxyitophene): poly (styrene sulfonate) (PEDOT: PSS) and a Pt electrode with a minimum detection limit of 1 μM (≃ 18•10^-3^ mg/dL) 159. In addition to being a valid alternative for glucose measurement, saliva can be used as a versatile biofluid for evaluating other clinically significant analytes (e.g., lactate and cholesterol). However, there are limitations in mouthguard devices as they are uncomfortable for long-term use, can lead to adverse effects related to dental conditions, are strictly site-specific, and the amount of these biofluids is limited 159-161.

### Glucose monitoring in tears

Several studies have suggested testing glucose in tear fluid as a blood substitute, thereby promoting the development of other techniques 162,163. If a good correlation between tear glucose and blood glucose can be established, the measurement of tear glucose levels could provide an interesting method of indirect measurement of blood glucose within normal, hyperglycemic, and hypoglycemic limits 164. To be analytically useful, sensing techniques require a very low detection limit (since glucose is present in tear fluid at levels 50-100 times lower than blood), high analytical sensitivity and selectivity, and the ability to quantitatively measure a very small sample in a short period of time (Figure 4E) 165. Yao et al. have integrated functional contact lenses consisting of a differential glucose biosensor module, metal interconnections, a readout circuit, an antenna, and a telecommunication circuit to monitor tear glucose levels wirelessly, continuously and non-invasively 165. The biosensor has a detection limit of 0.18 mg/dL and shows good linearity over the typical range of glucose concentrations in a tear film (0.18-10.5 mg/dL). Contact lenses can be worn for hours without discomfort and, therefore, provide an ideal vehicle for non-invasive and continuous glucose monitoring. However, a persistent problem for these types of devices is the implementation of a suitable power source. Recently, Badugu et al. introduced an optical chemical sensor for glucose detection in the ocular fluid 12. Scientific data evidenced how commercially available test strips exhibit the required performance to evaluate glucose concentration in low-volume tears 166-168. Reported data are summarized in Table 8 94,163-166.

### Glucose monitoring in urine

Urine is a noninvasive fluid for inexpensive, easy to use glucose testing, that has historically dominated diabetes monitoring before the blood glucose sensors era (Figure 4F) 169. However, as circulating glucose (up to modestly high level) is physiologically reabsorbed by the kidney 170, conditions of hypoglycemia, euglycemia and moderate hyperglycemia cannot be either identified and/or differentiated. Glycosuria is normally evidenced when the renal reabsorption threshold is exceeded (>180 mg/dL), even if there is individual variability 171,172. Thus, it is still of clinical interest for the evaluation of hyperglycemic episodes in poorly controlled patients, although there is no real-time relationship between glycosuria and blood glucose peaks. More recently, urine glucose determination may also help in the assessment of drug efficacy when using sodium-glucose cotransporter-2 (SGLT2) inhibitors, a class of oral anti-diabetic drugs that inhibit glucose reabsorption in the kidney, with elimination of glucose via urine 173. The evaluation of glycosuria should be performed only on fresh urine samples, since old or high bacterial load samples may alter the levels of glucose in urine. However, the lack of precision and sensitivity of this test limits its use 172, and as underlined by current literature in this field, efforts are in progress to provide more reliable glucose sensors for urine. To date, most of the proposed sensors are based on colorimetric approaches, as glycosuria is generally assessed by colorimetric strip-based devices, with enzymatic methods and chromogenic reagents 174. A recent, non-commercial alternative to colorimetric strips is represented by Surface-enhanced Raman scattering (SERS). This non-invasive sensing technique requires a very low sample volume (μL range) and no sample pre-processing, while it exhibits a high sensitivity and specificity for glucose 175,176. Reported data of urine-based glucose sensors are summarized in Table 9 174-179.

### Future directions

Glucose related sensing technologies are not novel, and relative devices have indeed required decades of evolution to become mature and established on the market. Currently, the evaluation of glucose levels in the blood or in other non-invasive fluids has evidenced the limits of the actual technology. Optical evaluation of glucose in ISF is inherently interfered by different molecules, such as lactate and urea, reducing the reliability of results. Also, studies have highlighted that glucose levels in the blood, as well as in ISF (and likely in other biofluids) are influenced by commonly used pharmacological treatments (e.g., acetaminophen, albuterol, lisinopril, atenolol, and atorvastatin). Besides, glucose oxidase and glucose dehydrogenase monitors should be avoided, respectively, in patients with abnormal oxygen saturation, and in peritoneal dialysis using icodextrin 100,180. Furthermore, even if non-invasive devices (such as those used for CGM) in some instances have replaced traditional glucometers, the detection of hypoglycemic events may be hampered by the time delay of glucose spread from blood to ISF. To overcome these drawbacks, current technology and methods should be improved, while other technological strategies should be searched for, and pursued. Barriers to glucose detection in non-invasive fluid sampling include analytical inaccuracy due to device miniaturization, the need to improve sensors' sensitivity and algorithms to convert sensor signals into glucose levels, in addition to a better understanding, by physicians, of the clinical significance of glucose values in non-blood fluids.

Another major point is to what extent glucose devices can be crucial to improve treatment and clinical outcomes. While devices for both SMBG and CGM have shown to be useful tools in type 1 diabetes with HbA1c and hypoglycemia as primary outcomes 108, devices for SMBG have not consistently shown a significant reduction in HbA1c in noninsulin treated patients with type 2 diabetes. On the other hand, in type 2 diabetes under insulin and/or hypoglycemic oral treatment, the use of a CGM device reduces HbA1c, but not the rates of hypoglycemia 108, while in gestational diabetes these devices help to achieve a better daytime glucose profile, providing an improvement in both maternal and neonatal outcomes 108, 181.

Despite the apparent success in both treatment and control of diabetes, in the last decade, however, a deterioration of glycemic control in US adults with diabetes has been reported 182, so that major efforts, also in the field of diabetes technology, should be undertaken to reverse these findings.

**Figure 5:**
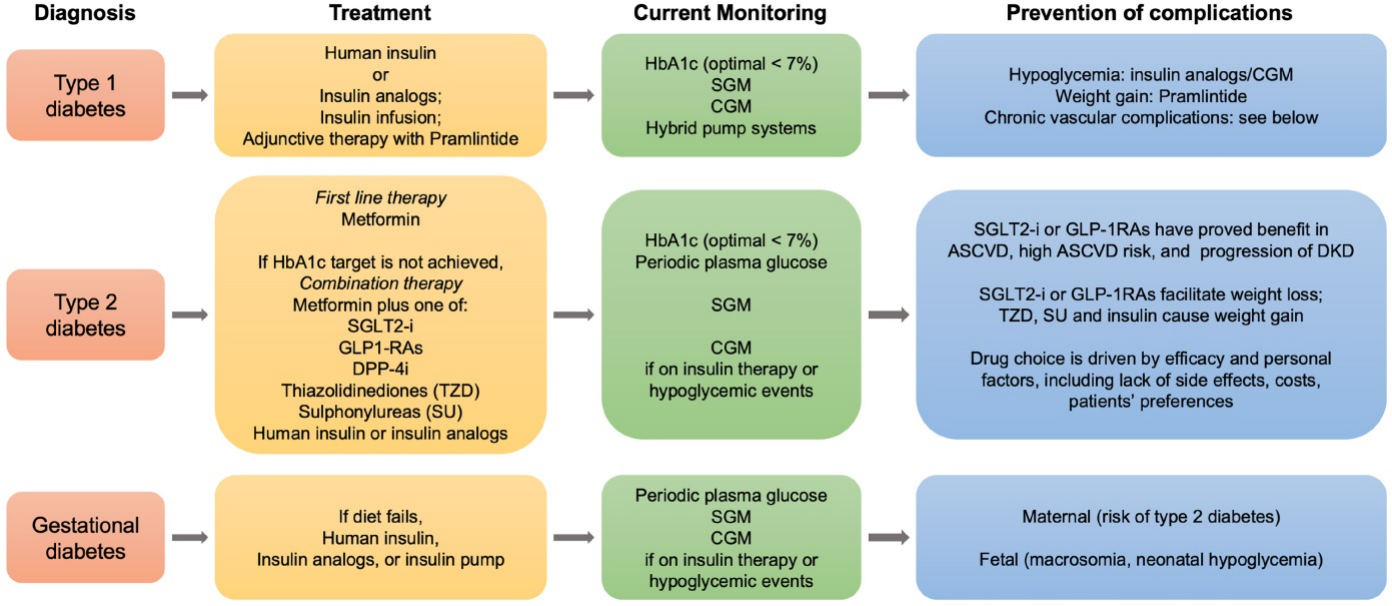
Roadmap for the management of diabetes mellitus. Summarized steps for the treatment and follow-up of type 1, type 2 and gestational diabetes are indicated. SGLT2, sodium glucose cotransporter 2; GLP-1RAs, glucagone-like peptide-1 receptor agonists; DPP-4i, dipeptydil peptidase-4 inhibitors; HbA1c, hemoglobin A1c; SGM, self glucose monitoring; CGM, continuous glucose monitoring; ASCVD, atherosclerotic cardiovascular disease; DKD, diabetic kidney disease.

As patients with diabetes experience the reduction of about a decade in their lifespan due to cardiovascular complications and renal failure, new therapeutic strategies have been developed and are in progress to ameliorate this outcome. In this context, the current roadmap for diabetes treatment, in particular for type 2 diabetes, has recently been revised by the ADA and the European Association for the Study of Diabetes, in light of the potential benefits of recently developed anti-diabetic drugs in preventing the vascular chronic complications linked to diabetes (Figure 5).

**Figure 6:**
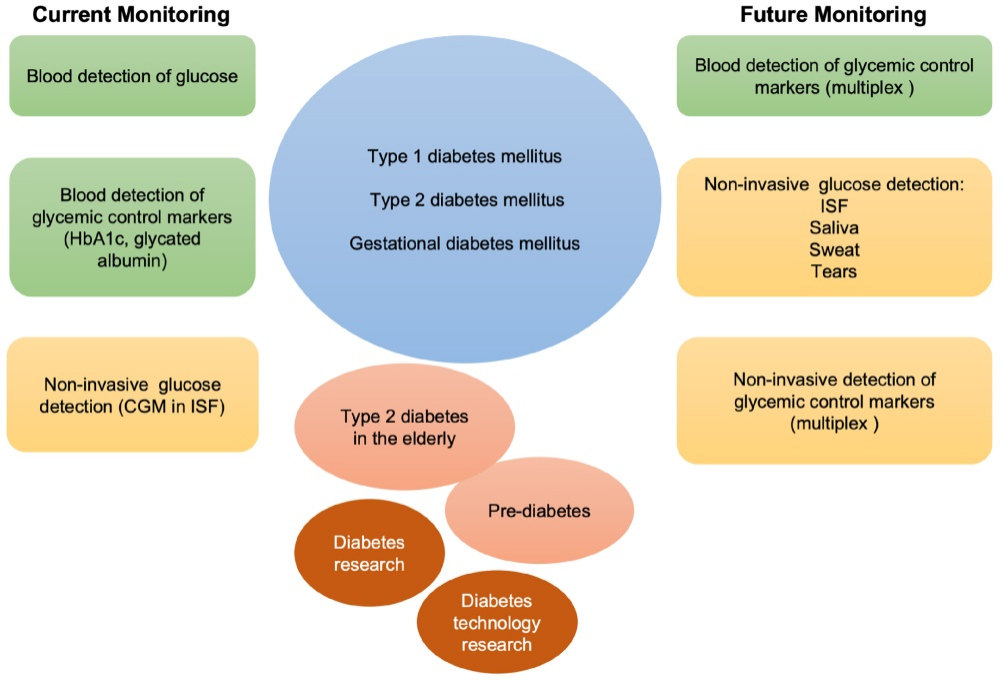
Schematic representation of current and future monitoring of glycemic control in type 1, type 2 and gestational diabetes. In green, blood is used for the detection of glucose and other markers of glycemic control. In orange, non-invasive biological fluids for glucose detection. Currently, the only approved FDA non-invasive methods for glucose detection employ ISF. Multiplex invasive or non-invasive assays may be foreseen in the future to integrate glucose measurement in the follow-up of patients with diabetes, elderly type 2 diabetes, and prediabetes, as well as to support diabetes related research.

Besides improving glycemic control and reducing hypoglycemic events, new single or combined pharmacological treatments may offer new tools potentially suitable for the clinical needs of diabetic patients. Adjunctive therapies in type 1 (insulin plus pramlintide, or SGLT2 inhibitors), and combination therapies in type 2 diabetes (metformin plus SGLT2 inhibitors or glucagon-like peptide analogs) may be compared with traditional treatments thanks to the use of conventional glucose devices and the introduction of new devices for CGM 183, 184. In this sense, technology well supports pharmacological research in diabetes and helps disentangling the pros and cons of each possible treatment. With the evolution of diabetes technology, non-invasive glucose monitoring has the potential to change the future of diabetes management 185, and to become soon, over the finger-pricking approaches, the preferred choice in young patients with type 1 diabetes, and in adults with prediabetes and overt type 2 diabetes (Figure 6).

Literature has evidenced novel and affordable biomarkers for the theranostics of diabetes 186-188, but current sensing technologies are poorly focused on such molecules, thus providing a bottleneck to the use of such biomarkers. Whether the development of lab-on-a chips may be useful to improve diagnostics, treatment, and clinical outcomes in diabetes is a big challenge that deserves further research.

## Conclusions

Overall, strategies to evolve the world of biosensors have been already undertaken by many researchers, and the results obtained bode well for future improvements both in device technology and in patients' lives. Even though blood-based glucose monitoring is still a gold standard technology, the exploitation of different biofluids for continuous glucose monitoring with no invasiveness may represent an attractive and promising option for future directions. Fundamental, in this case, is the development of specific biosensors. On the other hand, the important benefit of minimal invasiveness and continuous glucose monitoring of new medical devices may improve patient comfort and awareness of the glycemic status, with foreseeable improvements in clinical outcomes and, in general, with a positive impact in the healthcare system.

## Figures and Tables

**Table 1:** Representative international standards for assessment of acceptable performance

Regulatory Organism	Country	Device	Standard	Year	Ref.
Food and Drug Administration	USA	POCTs	FDA-2013-D-1445	2020	60
Food and Drug Administration	USA	OTC BGMS	FDA-2013-D-1446	2020	61
International Standard Organization	165 countries	BGMS	ISO 15197	2015	62-64
Clinical and Laboratory Standards Institute	>50 countries	POCTs	POCT12-A3	2018	62

POCT, point-of-care test; OCT, over-the-counter; BGMS, blood glucose monitoring test systems; FDA, food and drug administration; ISO, international standard organization.

**Table 2:** Classification of enzymatic glucose sensors

Classification	Characteristics
1st Generation	Based on the sensor designed by Clark and Lyons Formation of hydrogen peroxide Oxygen as an electron acceptor Errors due to interference from other electroactive species
2nd Generation	Replacement of oxygen as an electron acceptor Introduction of the non-physiological mediator Limitations in the transfer from the enzymatic active site to the electrode
3rd Generation	Absence of mediator Direct transfer between enzyme and electrode Low operating potential, higher selectivity, less interference

**Table 3:** Representative substrate enzyme immobilization method

Substrate	Enzyme	Immobilization
Au/Ag-NCs	GOx	Trapping
ITO/ Chitosan-Polypyrrole Au-NPs	GOx	Trapping
Ag/CNT/Chitosan	GOx	Layer technique
BDD/Graphene/ Pt-NPs	GOx	Absorption
Si/ VACNF	GOx	Absorption
Graphite NPs	GOx	Covalent bond

NC, nano cube; ITO, indium tin oxide; NP, nano particle; CNT, carbon nano tube; BDD, boron-doped diamond; VACNF, vertically aligned carbon nanofiber.

**Table 4:** Representative commercial and non-commercial glucose sensor for laboratory settings and SMBG

Manufacturer	Sample	Type	Sensor material	Method	Sensitivity	Linear Range (mg/dL)	LOD (mg/dL)
Commercial							
Roche Cobas 92 (laboratory)	S, P, U, CSF	Enzymatic (G6PD/NADP)	-	Photometric	1.003^§^	2-750	2
Roche Accu-check 93,94 (SMBG)	PB	Enzymatic (GDH/FAD)	Palladium	Electrochemical	0.127 μA/mM	10-600	10
Non-Commercial							
Yang et al. 95	Glc/NaOH*	Non-enzymatic	PDDA-graphene/CuO	Amperometric	4982.2 μA mM^-1^cm^-2^	0.072-72	0.004
Zang et al. 96	WB	GOx/HRP	TMB/GOx/HRP bi-enzymatic	Photometric	1.1 (a.u.)/(mg/dL)	49-284	5
Màrquez et al. 97	WB	Enzymatic (GOx/HRP)	Calcium alginate hydrogel	Amperometric	0.27 μA mM^-1^cm^-2^	36-218	0.007

S, serum; P, plasma; U, urine; CSF, cerebrospinal fluid; PB, peripheral blood; WB, whole blood; LOD, limit of detection; PDDA, poly-dimethyl diallyl ammonium chloride; TMB, 3,3', 5,5' tetramethylbenzidine dihydrochloride; HRP, horseradish peroxidase. ^§^angular coefficient provided by the linear regression (calibration curve y = 18.07·x-0.11 mg/dL). *Glucose in alkaline solution.

**Table 5:** Main technologies and characteristics of the glucose evaluation in ISF

Technologies	Type	Advantages	Disadvantages	Measurement site	Performance	Ref.
Electrical	Impedance Spectroscopy	It can measure glucose levels in the vascular compartment, so no time lag in sensor response Low-cost instrument	Temperature and diseases affecting skin may affect measurements Changes in blood dielectric properties may cause error in measurement	Skin, wrist	Sens: 0.02-0.05 Ω/(mg/dL)	103, 104
Optical	Raman	Non-invasive sharp spectral features Broad SERS substrate enhancement	Weak Raman signal Background noise	Wrist, finger, aqueous humor		110-114
Optical	OCT	Real time monitoring High resolution High signal to noise ratio High penetration depth Robust to blood pressure, heart rate, and haematocrit variability.	sensitive to motion artifacts sensitive to large changes in temperature	Forearm	Sens. 0.015-0.045 a.u./(mg/dL)	117,118,123
Optical/Mechanical	Photoacoustic spectroscopy	It is not affected by ionic strength. Higher sensitivity	Problem of scattering in the tissues Sensitive to environmental parameters, chemical interferences from other metabolites, physical interference from temperature and pressure changes.	Aqueous humor, finger and forearm	Sens. 0.035-0.098 /100(mg/dL)	118,124
Optical	Fluorescence	No damage to the body Information about the structure and micro-environment of molecules	Scattering phenomena Fluorescence can depend on skin pigmentation, redness, epidermal thickness	Skin	Range: up to 454 mg/dL	119
Optical	NIR	Skin penetration up to 1-100 mm High sensitivity Low cost and widespread	Poor signal to noise ratio Calibration issues Baseline drift Thermal noise Physiological factors Environmental factors	Tongue, oral mucosa, lip, ear lobe, finger, forearm, cheek.	Range:30-300 mg/dL and up to 600 mg/dL	125-143
Electrochemical	Iontophoresis	No mechanical hardware Simpler concept	Filtered ISF and thus more similar to sweat or saliva Skin irritation over long term Onset sweating	Skin, wrist, forearm		144
Microwave		No-ISF extraction is required Flexible substrate	Lower precision	Skin	Range: 36-454 mg/dL	145

**Table 6:** Representative characteristics of non-commercial sweat-based glucose sensors

Manufacturer		Type	Sensor material	Configuration	Sensitivity	Linear range (mg/dL)	LOD (mg/dL)
Lee et al. 18		GOx	Au/Nafion/Glutaraldehyde	Amperometric	28 μA mM^-1^cm^-2^	0-18	0.2
Gao et al. 148		GOx	Chitosan/CNT /Prussian blue/Au	Amperometric	2.35 nA μM^-1^	0-3.6	-
	Saraoğlu et al. 147	Non-enzymatic (humidity)	Thin film	Capacitive- ANN	-	83-116.5	-
	Lu et al. 152	Non-enzymatic	Chitosan/NiCo_2_O_4_/Au	Amperometric	0.5 μA μM^-1^	0.2-4	0.2

**Table 7:** Representative commercial and non-commercial characteristics of saliva-based glucose sensors

Manufacturer		Type	Sensor material	Configuration	Sensitivity	Linear Range (mg/dL)	LOD (mg/dL)
Commercial							
The IQ Global Group 156		GOx	Organic Transistor ITO/P3HT/Poly (4-vinylphenol)	Amperometric	-	3.6-545	-
Non-commercial							
Macaya et al. 155		GOx	Pt/PEDOT:PSS	Resistive	0.1 R/R_0_/(mg/dL)	0.02-545	0.02
Chakraborty et al. 157		Non-enzymatic	porous CuO	Amperometric	∼2299 μAmM^-1^ cm^-2^	0.09-4	0.008
	Liu et al. 158	GOx/HRP	MWCNT	Amperometric	67.93 nAmM^-1^	0.9-27	0.005
							

P3HT, poly(3-hexylthiophene); R/R_0_, relative resistance variation; PEDOT:PSS, poly(3,4-ethylenedioxythiophene) polystyrene sulfonate; MWCNT, multi-walled carbon nanotube.

**Table 8:** Representative commercial and non-commercial characteristics of tears-based glucose sensors

Manufacturer	Type	Sensor material	Configuration	Sensitivity	Linear Range (mg/dL)	LOD (mg/dL)
Commercial						
Roche - ACCU-CHEK Aviva Plus 94,163	PQQ-GDH	nitrosoaniline-derivative (Mediator)	Amperometric	0.127 μAmM^-1^	0.009-2.67	0.016
Non-Commercial						
Kownacka et al. 164	GOx	Pt/Ir	Amperometric	-	1.8-18	-
Kim et al. 165	Non-enzymatic	Nanoparticle Embedded Contact Lens	Photometric	0.089Δr^'^_n_/mM	0-44	-
Romeo et al. 166	Non-enzymatic	PET/Au/CuO/Nafion	Amperometric	850 μAmM^-1^ cm^-2^	0.055-12.6	0.05

PQQ-GDH, pyrroloquinoline quinone dehydrogenase; PET, polyethylene terephthalate; Δr^'^_n_, difference of relative reflectance before and after reaction with glucose.

**Table 9:** Representative commercial and non-commercial characteristics of urine-based glucose sensors

Manufacturer	Type	Sensor material	Configuration	Sensitivity	Range (mg/dL)	LOD (mg/dL)
Commercial						
Sysmex, UC‐11A test strips 174	GOx	-	Colorimetric (Semiquantitative)	-	50-2000	50
Non-Commercial						
Kong et al. 175,176	SERS	Metal carbonyl compounds	Raman responses	1800-2200 cm^-1^	1.8-180	1.8
Lee et al. 177	GOx	Paper/ PAni-NPs/RBCM	Colorimetric	0.2562 λ/(μg/mL)	0-1018	10
Janyasupab et al. 178	Non-enzymatic	CoFe/NG	Amperometric	45.36 μAmM^-1^ cm^-2^	5-55	∼4
Sun et al. 179	Non-enzymatic	Cu-MOF	Amperometric	89 μAmM^-1^ cm^-2^	0.001-90	0.2·10^-3^

SERS, surface-enhanced Raman scattering; PAni-NPs, polyaniline-nanoparticles; RBCM, red blood cell membrane; NG, nitrogen-doped graphene; MOF, metal-organic framework. λ, absorbance at 563 nm.
